# FK-16 Derived from the Anticancer Peptide LL-37 Induces Caspase-Independent Apoptosis and Autophagic Cell Death in Colon Cancer Cells

**DOI:** 10.1371/journal.pone.0063641

**Published:** 2013-05-20

**Authors:** Shun X. Ren, Jin Shen, Alfred S. L. Cheng, Lan Lu, Ruby L. Y. Chan, Zhi J. Li, Xiao J. Wang, Clover C. M. Wong, Lin Zhang, Simon S. M. Ng, Franky L. Chan, Francis K. L. Chan, Jun Yu, Joseph J. Y. Sung, William K. K. Wu, Chi H. Cho

**Affiliations:** 1 School of Biomedical Sciences, The Chinese University of Hong Kong, Hong Kong, China; 2 Institute of Digestive Disease, Li Ka Shing Institute of Health and Department of Medicine and Therapeutics, The Chinese University of Hong Kong, Hong Kong, China; 3 Department of Surgery, The Chinese University of Hong Kong, Hong Kong, China; Southern Illinois University School of Medicine, United States of America

## Abstract

Host immune peptides, including cathelicidins, have been reported to possess anticancer properties. We previously reported that LL-37, the only cathelicidin in humans, suppresses the development of colon cancer. In this study, the potential anticancer effect of FK-16, a fragment of LL-37 corresponding to residues 17 to 32, on cultured colon cancer cells was evaluated. FK-16 induced a unique pattern of cell death, marked by concurrent activation of caspase-independent apoptosis and autophagy. The former was mediated by the nuclear translocation of AIF and EndoG whereas the latter was characterized by enhanced expression of LC3-I/II, Atg5 and Atg7 and increased formation of LC3-positive autophagosomes. Knockdown of Atg5 or Atg7 attenuated the cytotoxicity of FK-16, indicating FK-16-induced autophagy was pro-death in nature. Mechanistically, FK-16 activated nuclear p53 to upregulate Bax and downregulate Bcl-2. Knockdown of p53, genetic ablation of Bax, or overexpression of Bcl-2 reversed FK-16-induced apoptosis and autophagy. Importantly, abolition of AIF/EndoG-dependent apoptosis enhanced FK-16-induced autophagy while abolition of autophagy augmented FK-16-induced AIF−/EndoG-dependent apoptosis. Collectively, FK-16 induces caspase-independent apoptosis and autophagy through the common p53-Bcl-2/Bax cascade in colon cancer cells. Our study also uncovered previously unknown reciprocal regulation between these two cell death pathways.

## Introduction

Antimicrobial peptides (AMPs), also known as host defense peptides, exist in eukaryotic cells as a conserved component of the innate immune system. AMPs perform first-line defense against infection by acting as “natural antibiotics” by direct killing of pathogenic microbes [1], [2]. The selectivity of AMPs to bacterial cells relies on their cationic structures that are crucial for the interaction with negatively charged bacterial membranes [3], [4]. Emerging evidence suggests that AMPs may also selectively bind to cancer cells over untransformed cells because of the increased surface exposure of negatively charged phosphatidylserine in cancer [5]. Increased levels of O-glycosylated mucins, negative membrane potential, and increased membrane fluidity and cell-surface area in cancer cells may also contribute to this selectivity [6]. Numerous AMPs of human (e.g. β-definsin, LL-37) and non-human (e.g. BMAP-28, lactoferricin B, magainin II, melittin, tachyplesin I) origins have been demonstrated to exert cytotoxicity on cancer cells through diverse mechanisms [6]. For instance, bovine lactoferricin B induced mitochondrial pathway of apoptosis in human leukemia and carcinoma cell lines but not untransformed cells through generation of reactive oxygen species [7]. Magainin II also induced cell death in bladder cancer cells but not normal fibroblasts through pore formation on cell membrane and subsequent cell lysis [8]. Human β-definsin-1, which exhibited cancer-specific loss of expression in renal clear cell carcinoma, induced caspase-3-mediated apoptosis in the renal cancer cell line SW156 [9]. According to the AMP database (http://aps.unmc.edu/AP/main.php), over 130 such peptides are known to have anticancer properties [10].

Cathelicidins are a family of evolutionarily conserved AMPs. hCAP-18 is the only cathelicidin in humans. This 18-kDa preproprotein consists of an N-terminal signal sequence, a cathelin-like domain, and a C-terminal AMP domain. Proteolytic cleavage of hCAP-18 releases a 37-residue, amphipathic, helical peptide known as LL-37. This peptide is secreted by bone marrow cells, circulating leukocytes, and numerous types of epithelial tissues, such as skin and gastrointestinal mucosa. LL-37 not only exhibits a board spectrum of antimicrobial activities (e.g. bacteria, fungi, and viruses), but also has the ability to neutralize bacterial lipopolysaccharides. Importantly, LL-37 could mediate innate immunity through regulating chemotaxis of leukocytes (e.g. neutrophils, monocytes, T-cells, eosinophils and mast cells) and production of cytokines at sites of infection and inflammation as well as promoting re-epithelization during wound healing [11], [12], [13], [14]. Cumulative evidence from tumor biology studies indicates that LL-37 plays a prominent role in carcinogenesis [15]. For instance, LL-37 exerts anticancer effects on gastric cancer and T leukemic cells [16], [17]. The C-terminal fragment of LL-37 also exhibits cytotoxicity towards both drug-resistant and drug-sensitive oral epitheloid carcinoma cells [18]. Our previous study also showed that the expression of LL-37 was remarkably downregulated in human colon cancer tissues whereas exogenous LL-37 induced apoptotic cell death in cultured colon cancer cells. Importantly, cathelicidin-deficient mice exhibited increased susceptibility to azoxymethane-induced colon carcinogenesis [19]. These findings suggest that LL-37 is an endogenous tumor-suppressing peptide.

Given its importance in immunology and cancer research, efforts have been put forth to study the structural and biophysical properties of LL-37. Moon *et al.* first described the use of glutathione S-transferase fusion system for the expression and purification of isotope-labeled LL-37 in *Escherichia coil* [20]. Structural analysis by NMR spectroscopy demonstrated that LL-37 is characterized by a long amphipathic helix spanning residues 2–31 with the whole molecule curved a train of hydrophobic side chains [21]. Ramamoorthy *et al.* showed that LL-37 orients near the surface of phospholipid bilayers and forms oligomeric structures [22]. To this end, LL-37 possesses the ability to disrupt cell membrane [23], [24], [25].

The cost associated with chemical synthesis is one of the limiting factors that hamper the use of peptides as therapeutic agents. It is therefore important to identify the functional region of LL-37 so that shorter fragments which retain the biological activity of the full-length peptide can be produced with lower cost. Previous structure-function analysis of different LL-37 fragments has demonstrated that LL7-27, a 21-residue peptide derived from residues 7–27 of LL-37, exhibits potent activity against microbes (particularly Gram-positive bacteria) through disruption of the lipid bilayer structure [26]. Another study showed that the N-terminal fragment LL-12 corresponding to residues 1–12 of LL-37 is inactive against bacteria or cancer cells whereas FK-16 corresponding to residues 17–32 retains antibacterial and antitumor effects [27]. This fragment even has a better activity against prokaryotes and nucleated cells than the full-length peptide [28], suggesting that FK-16 may be a promising candidate for further characterization as a novel anticancer peptide. In the present study, the *in vitro* anticancer effect of FK-16 was investigated.

## Results

### FK-16 versus LL-37 in the Induction of Cell Death in Colon Cancer Cells

**Figure 1:**
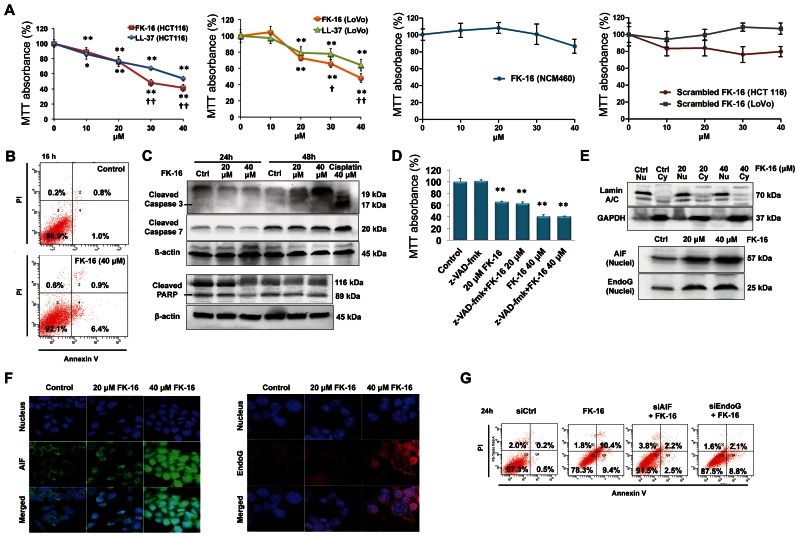
Induction of caspase-independent but AIF/EndoG-dependent apoptosis by the anticancer peptide FK-16 in colon cancer cells. (**A**) Effects of LL-37, FK-16, and scrambled FK-16 (48 h) on viability of colon cancer cells (HCT116, LoVo) were determined by MTT assay. The cytotoxic effect of FK-16 was also assayed in human normal colon mucosal epithelial NCM460 cells. (**B**) Phosphotidylserine externalization in HCT116 cells treated with or without FK-16 was determined by flow cytometry of PI/Annexin V-stained cells. (**C**) Effects of FK-16 on PARP cleavage and caspase activation in HCT116 cells were determined by Western blot. Cisplatin was used as a positive control for caspase activation. (**D**) Pre-treating HCT116 cells with the pan-caspase inhibitor z-VAD-fmk for 1 h did not prevent FK-16-induced loss of cell viability as determined by MTT assay (24 h). (**E**) Nuclear expression of AIF and EndoG in HCT116 cells were analyzed by Western blot analysis of fractionated proteins. GAPDH and Lamin A/C were used as internal controls for cytosolic (Cy) and nuclear (Nu) proteins, respectively. (**F**) Effects of FK-16 (6 h) on subcellular localization of AIF (green) and EndoG (red) in HCT116 were determined by confocal immunofluorescence (400×). (**G**) Knockdown of AIF and EndoG partially reversed phosphotidylserine externalization induced by FK-16 (40 µM; 24 h) in HCT116. Cells were challenged with FK-16 at 48 h post-transfection of AIF- and EndoG-siRNAs. Data were presented as mean ± SD from three separate experiments. *, *p*<0.05; **, *p*<0.01, significantly different from the respective control group.

The cytotoxicities of FK-16 and the full-length LL-37 were initially investigated in two human colon cancer cell lines (LoVo and HCT116) by MTT assay. As shown in Fig. 1A, both FK-16 and LL-37 significantly reduced the viability of LoVo and HCT116 cells in a dose-dependent manner. Noticeably, FK-16 displayed a better activity against colon cancer cells than LL-37. Moreover, FK-16 had a minimal effect on the viability of human normal colon mucosal epithelial NCM460 cells. A control peptide with scrambled sequence of FK-16 also had negligible effects on LoVo and HCT116, indicating FK-16 mediated selective killing of cancer cells. As HCT116 was more sensitive than LoVo to FK-16, HCT116 was selected for subsequent mechanistic study and treated with either 20 µM or 40 µM FK-16, at which ∼20% and ∼50% of cytotoxicity occurred.

### Induction of AIF/EndoG-dependent Apoptosis by FK-16

We have previously shown that LL-37 could induce caspase-independent apoptosis in colon cancer cells [19]. To determine if apoptosis mediated the cytotoxicity of FK-16, the loss of phosphatidylserine asymmetry, which is a hallmark of apoptosis, was quantified by cytometry of propidium iodide/Annexin V double-stained cells. As shown in Fig. 1B, FK-16 induced phosphatidylserine externalization without increasing the proportion of propidium iodide^+^ cells, indicating that FK-16 induced apoptosis but not necrotic cell death. At both 24 h and 48 h time-points, FK-16 treatment did not increase PARP cleavage nor activate caspases-3 and 7 (Fig. 1C). Concordantly, the pan-caspase inhibitor z-VAD-fmk failed to reverse the loss of cell viability caused by FK-16 (Fig. 1D). These findings suggested that FK-16 induced apoptosis in a caspase-independent manner. AIF and EndoG, originally localized in the mitochondria and translocated into the nucleus upon activation, are known mediators of caspase-independent apoptosis [29]. Immunoblotting using fractionated protein lysates demonstrated that FK-16 increased the nuclear levels of AIF and EndoG (Fig. 1E). Immunofluorescence confirmed that AIF and EndoG redistributed from the cytosol to the nucleus in FK-16-treated HCT116 cells (Fig. 1F). The functional involvement of AIF and EndoG in FK-16-induced apoptosis was further verified by RNA interference experiments, in which knockdown of AIF or EndoG attenuated phosphatidylserine externalization induced by FK-16 (Fig. 1G). These findings indicated that, similar to LL-37, FK-16 induced AIF/EndoG-dependent but caspase-independent apoptosis in colon cancer cells.

### Induction of Autophagic Cell Death by FK-16 but not LL-37

**Figure 2:**
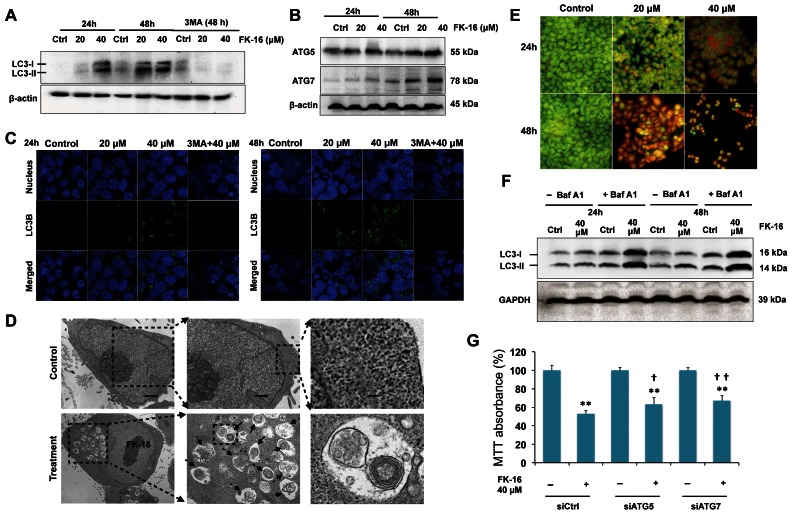
Induction of autophagic cell death by FK-16 in colon cancer cells. (**A**) HCT116 cells exhibited increased protein expression of LC3-I/II after treatment with FK-16 for 24 h and 48 h. LC3 expression induced by FK-16 was abolished by the autophagy inhibitor 3-MA (5 mM, 1 h pre-treatment). (**B**) FK-16 also induced Atg5 and Atg7 protein expression. (**C**) Treating HCT116 with FK-16 for 24 h or 48 h prominently enhanced the formation of autophagic vacuoles as determined by immunofluorescent staining for LC3 (400×). The formation of LC3^+^ autophagosomes induced by FK-16 was blocked by 3-MA. (**D**) Ultrastructural analysis by electron microscopy revealed the formation of autophagosome or secondary lysosomes with the residual digested material in FK-16-treated HCT116 cells (40 µM; 48 h). (**E**) The accumulation of acidic vesicular organelles, which emitted bright red fluorescence, induced by FK-16 (40 µM) was visualized by acridine orange staining (400×). (**F**) Increased autophagic flux was confirmed by co-treating HCT116 cells with FK-16 and bafilomycin A_1_. Treatment with bafilomycin A_1_ (10 nmol/L) did not prevent the upregulation of LC3-I/II in cells incubated with FK-16 (40 µM). (**G**) Knockdown of Atg5 or Atg7 attenuated the cytotoxic effect of 48-h treatment of FK-16 (40 µM) in HCT116. Data are presented as means ± S.D. of three separate experiments. *, *p*<0.05; **, *p*<0.01 significantly different from the respective control group. **^†^**, *p*<0.05; **^††^**, *p*<0.01 significantly different from control siRNA-transfected cells treated with FK-16.

To determine the effect of FK-16 on another caspase-independent cell death pathway, namely, autophagic cell death [30], we analyzed the expression of LC3 protein (particularly LC3-II which is known to correlate with the extent of autophagy) and two other autophagy-related proteins, i.e. Atg5 and Atg7. Results showed that FK-16 increased LC3-I and LC3-II as well as Atg5 and Atg7 protein expression (Fig. 2A & 2B). FK-16 also induced the formation of LC3^+^ autophagic vacuoles in HCT116 (Fig. 2C). Both the induction of LC3-I/II expression and the formation of LC3^+^ autophagic vacuoles by FK-16 could be blocked by 3-methyladenine, a Class III phosphoinositide 3-kinase inhibitor. By contrast, the full-length LL-37 peptide had minimal effect on LC3-I/II expression (data not shown). Ultrastructural analysis by electron microscopy revealed that a 48 h-exposure to FK-16 induced massive vacuolization in HCT116 cells, in which lamellar and myelin-like structures resembling late autophagic vacuoles were observed (Fig. 2D). Formation of acidic vesicular organelles, which is an important hallmark of autophagy, was also enhanced by FK-16 as determined by vital staining of HCT116 cells with acridine orange, a dye that emits bright red fluorescence in acidic vesicles but fluoresces green in cytoplasm and nucleus (Fig. 2E). The formation of autolysosomes was also increased in FK-16-treated cells as visualized by monodansylcadaverine staining (data not shown). The increase in autophagic flux caused by FK-16 was confirmed by treating HCT116 cells with FK-16 and bafilomycin A_1_ (a lysosomotropic agent), alone or in combination. Inhibition of lysosomal function by bafilomycin A_1_ increased the levels of both LC3-I and -II induced by FK-16 (Fig. 2F), suggesting that FK-16 increased autophagic flux. Depending on cellular context, autophagy may serve as a pro-death or pro-survival mechanism. To determine the functional role of autophagy induced by FK-16, a RNA interference approach was used to abolish autophagy by targeting Atg5 and Atg7, both of which are required for the formation of autophagosomes at the early stage. Knockdown of Atg5 or Atg7 significantly reduced the cytotoxic effect of FK-16 (Fig. 2G), suggesting that FK-16 induced autophagic cell death in colon cancer cells.

### Requirement of p53 for FK-16-induced Apoptosis and Autophagic Cell Death

**Figure 3:**
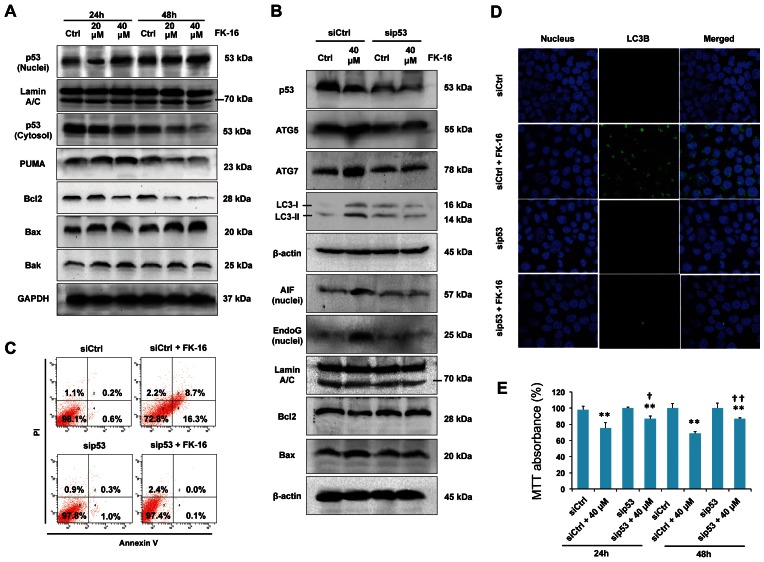
Activation of p53 was required for AIF/EndoG-dependent apoptosis and autophagic cell death induced by FK-16. (**A**) HCT116 cells were incubated with FK-16 for 24 h or 48 h. Cytosolic and nuclear p53 and total expression of Bcl-2 members (PUMA, Bcl-2, Bax and Bak) were determined by Western blot. GAPDH and Lamin A/C were used as internal controls for cytosolic and nuclear proteins, respectively. (**B**) Knockdown of p53 reversed the upregulation of pro-autophagic factors (Atg5 and Atg7) and pro-apoptotic factors (Bax, Bak, nuclear AIF and nuclear EndoG) as well as downregulation of Bcl-2 by FK-16. (**C**) FK-16 failed to induced phosphotidylserine externalization in p53-depleted HCT116 cells. After transfection with control- or p53-siRNA for 48 h, cells were treated with or without FK-16 (40 µM) for another 24 h followed by propidium iodide/annexin V-double staining. (**D**) Knockdown of 53 markedly reduced the number of LC3^+^ autophagic vacuoles in FK-16-treated cells (40 µM; 48 h) as determined by confocal immunofluorescence (400×). Nuclei (blue) were stained with DAPI. (**E**) Knockdown of p53 partially reversed the inhibitory effect of FK-16 on cell viability in HCT116 as determined by MTT assay. Data are presented as means ± S.D. of three separate experiments. *, *p*<0.05; **, *p*<0.01 significantly different from the respective control group. **^†^**, *p*<0.05; **^††^**, *p*<0.01 significantly different from control siRNA-transfected cells treated with FK-16.

The tumor suppressor protein p53 is known to take part in both caspase-dependent and -independent cell death, including AIF/Endo-dependent apoptosis and autophagy [31], [32]. Here, we demonstrated that FK-16 directly activated p53 to mediate both cell death pathways. As shown in Fig. 3A, FK-16 increased the nuclear level of p53 and upregulated the expression of PUMA and Bax. Consistently, FK-16 reduced the expression of Bcl-2. To elucidate the functional involvement of p53 in AIF/Endo-dependent apoptosis and autophagic cell death triggered by FK-16, p53 was knocked down by siRNA. Knockdown of p53 not only restored the expression of Bax and Bcl-2, but also remarkably reduced FK-16-induced LC3-I/II, Atg5 and Atg7 expression as well as nuclear expression of AIF and EndoG, suggesting that p53 was a common activator of both cell death pathways (Fig. 3B). Accordingly, knockdown of p53 reduced phosphatidylserine externalization (Fig. 3C), the formation of LC3^+^ autophagic vacuole (Fig. 3D) and the loss of cell viability (Fig. 3E) induced by FK-16.

### Altered Expression of Bcl-2 and Bax during FK-16-induced Apoptosis and Autophagic Cell Death

**Figure 4:**
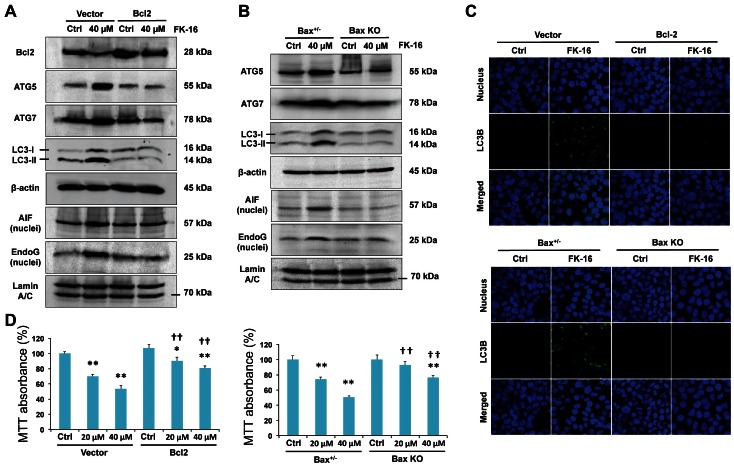
Altered expression of Bcl-2 and Bax was required for AIF/EndoG-dependent apoptosis and autophagic cell death induced by FK-16. (**A**) Restoration of Bcl-2 expression by transfection with Bcl-2-encoding plasmid reversed the upregulation of pro-autophagic (Atg5, Atg7 and LC3-I/II) and pro-apoptotic (nuclear AIF and nuclear EndoG) factors induced by FK-16 (40 µM; 48 h) in HCT116. (**B**) Genetic ablation of Bax (Bax KO) in HCT116 reversed FK-16-induced apoptotic and autophagic signals. Bax^+/−^ HCT116 treated with or without FK-16 was used as control. (**C**) Restoration of Bcl-2 expression or genetic ablation of Bax in HCT116 abolished the formation of LC3^+^ autophagic vacuoles induced by FK-16 (40 µM; 48 h) as determined by confocal immunofluorescence (400×). (**D**) Restoration of Bcl-2 expression or genetic ablation of Bax partially reversed the inhibitory effect of FK-16 on cell viability in HCT116 as determined by MTT assay. Data are presented as means ± S.D. of three separate experiments. *, *p*<0.05; **, *p*<0.01 significantly different from the respective control group. **^††^**, *p*<0.01 significantly different from empty vector-transfected or Bax^+/−^ cells treated with FK-16.

Since the Bcl-2 family has been reported to regulate both caspase-independent apoptosis and autophagy in other biological contexts [30], [32], we next questioned if FK-16-induced colon cancer cell death was mediated through Bcl-2 and Bax, both of which were downstream of p53 (Fig. 3B). Results showed that restoration of Bcl-2 expression by transfection with Bcl-2-encoding plasmid or genetic ablation of Bax using Bax^−/−^ HCT116 cells abolished FK-16-induced expression of LC3-I/II, Atg5 and Atg7 as well as nuclear expression of AIF and EndoG (Fig. 4A & B), suggesting that downregulation of Bcl-2 and upregulation of Bax were required for FK-16-induced autophagic and apoptotic signaling. In line with these findings, restoration of Bcl-2 expression or genetic ablation of Bax reduced the formation of LC3^+^ autophagic vacuole (Fig. 4C) and the loss of cell viability (Fig. 4D) induced by FK-16. These findings indicate that FK-16 acted through the p53-Bcl-2/Bax pathway to simultaneously induce AIF/EndoG-dependent apoptosis and autophagy-mediated cell death.

### Reciprocal Regulation between Caspase-independent Apoptosis and Autophagic Cell Death

**Figure 5:**
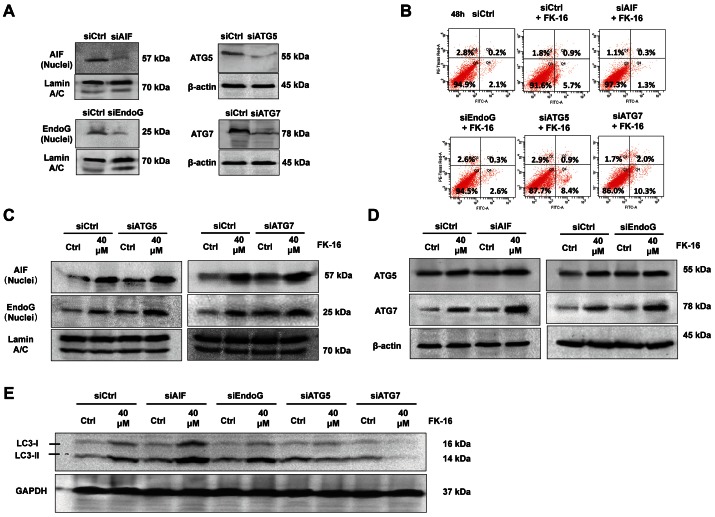
Reciprocal regulation of AIF/EndoG-dependent apoptosis and autophagic cell death induced by FK-16. (**A**) The knockdown efficacies of AIF-, EndoG-, Atg5- and Atg7-siRNAs were confirmed by downregulation of protein levels of respective targets in HCT116. (**B**) Knockdown of Atg5 or Atg7 enhanced basal and FK-16-induced (40 µM; 6 h) nuclear expression of AIF and EndoG. (**C**) Knockdown of Atg5 or Atg7 increased FK-16-induced (40 µM; 48 h) phosphotidylserine externalization as determined by annexin V staining and flow cytometry. (**D**) Knockdown of AIF or EndoG enhanced basal and FK-16-induced (40 µM; 48 h) Atg5 and Atg7 protein expression. (**E**) Knockdown of Atg5 or Atg7 abolished whereas Knockdown of AIF or EndoG enhanced FK-16-induced (40 µM; 48 h) LC3-I/II expression in HCT116. Data are representative of three independent experiments.

To elucidate the potential crosstalk between autophagic cell death and AIF/EndoG-dependent apoptosis in the action of FK-16, these two cellular processes were abolished by RNA interference. The knockdown efficacies of Atg5-, Atg7-, AIF- and EndoG-siRNAs were confirmed by Western blot (Fig. 5A). Inhibition of autophagy by Atg5- or Atg7-siRNA substantially augmented nuclear expression of AIF and EndoG induced by FK-16 (Fig. 5B). Concordantly, knockdown of Atg5 or Atg7 also enhanced FK-16-induced phosphatidylserine externalization (Fig. 5C), suggesting that inhibition of autophagy intensified FK-16-induced AIF/EndoG-dependent apoptosis. Reciprocally, abolition of AIF/EndoG-dependent apoptosis by AIF- or EndoG-siRNA enhanced Atg5 and Atg7 expression induced by FK-16 (Fig. 5D), indicating that inhibition of AIF/EndoG-dependent apoptosis magnified the autophagic signal in FK-16-treated colon cancer cells. Concordantly, AIF- and EndoG-siRNAs enhanced the expression of LC3-I/II. These findings indicated the existence of an unreported reciprocal regulation between autophagic cell death and AIF/EndoG-dependent apoptosis.

## Discussion

Induction of apoptosis in tumor cells has long been recognized as an essential approach for cancer therapy [33]. However, the intrinsic resistance of some tumor cells to apoptosis and the rapid regrowth of tumor after initial response to chemotherapeutic agents remain major clinical conundrums. Therefore, there is an increasing interest within the scientific community in studying the full gamut of caspase-independent cell death and identifying agents that act primarily through the caspase-independent mechanism. In the present study, we demonstrate that treatment with the anticancer peptide FK-16 strongly reduced the viability of colon cancer cells in the absence of caspase activation or membrane permeabilization, suggesting that neither the classical caspase-dependent apoptosis nor necrosis was responsible for its cytotoxicity. To this end, FK-16-treated cells exhibited biochemical and morphological features consistent with AIF/EndoG-dependent apoptosis and autophagic cell death. Above all, knockdown of genes central to these two cellular processes attenuated the cytotoxicity of FK-16. These findings suggest that FK-16 is a dual-mode activator of caspase-independent cell death. However, it is also worthwhile to notice that in some experimental settings (Fig. 1G & 3C), FK-16 modestly increased propidium iodide staining. This observation might be explained by the heightened cellular stress associated with siRNA transfection, which could render the cells more susceptible to the membrane-disrupting effect of FK-16.

A number of cellular stresses, including DNA damage and oncogene activation, have been shown to activate p53. Depending on the initial stimulus and the cellular context, p53 activation could trigger a repertoire of responses, including cell cycle arrest, apoptosis, cellular senescence, differentiation and autophagy [34]. Since the loss of p53 expression is common in human cancers, restoring p53 activity is an attractive approach for cancer therapy. In this respect, a number of p53-restoring experimental anticancer agents (e.g. CP-31398 and Nutlin) have been identified [35]. Here we show that FK-16 is a novel activator of p53 signaling. FK-16 not only increased nuclear p53 accumulation, but also induced the expression of PUMA and Bax, both of which are known transcriptional targets of p53 [36], [37]. Knockdown of p53 also reversed FK-16-induced AIF/EndoG-dependent apoptosis and autophagic cell death. Moreover, the pro-apoptotic and pro-autophagic effect of FK-16 required p53-dependent upregulation of Bax and downregulation of Bcl-2. In lines with these findings, the central roles of the p53-Bax/Bcl-2 cascade in AIF/EndoG-dependent apoptosis and autophagy have been reported. For instance, an analog of the DNA-damaging drug cyclophosphamide and a major green tea polyphenol have been shown to activate p53 and Bax to trigger nuclear relocation of AIF and EndoG to mediate apoptosis in tumor cells [32], [38]. p53 as a transcription factor also stimulates autophagy through inducing multiple pro-autophagic genes, including *DRAM*, *SKP2*, *SESN2*, and *ULK1/2*, in addition to *PUMA* and *BAX* [30]. Moreover, p53 activation could promote the dissociation of the Bcl-2-Beclin-1 complex and thereby disinhibiting Beclin-1-dependent autophagy [39].

An interesting finding emerging from this work is the discovery of a reciprocal regulatory mechanism between the two caspase-independent cell death pathways. Our data indicates that abolition of AIF/EndoG-dependent apoptosis enhances autophagic cell death and vice versa, indicating that these two pathways mediate the cytotoxicity of FK-16 in a cooperative manner. Although FK-16-induced autophagy is found to be pro-death in nature, blockade of autophagy paradoxically induces caspase-independent apoptosis in FK-16-treated cells. An explanation to this conundrum is that blockade of FK-16-induced autophagy prevents cell death but still activates caspase-independent apoptosis in a compensatory manner and the net outcome is the preservation of cell viability as compared with cells in which both cell death pathways are activated. To this end, the direct contribution of autophagy to cell death has been widely reported despite the recent disputes over actual existence of “autophagic cell death” [40], [41], [42], [43]. While co-activation of autophagy and caspase-independent apoptosis has been documented in various biological contexts [44], [45], [46], there are only sporadic studies in the literature that have tried to elucidate the relationship between these two processes. Consistent with our findings, Zheng *et al.* and Eimer *et al.* found that autophagy could protect against caspase-independent apoptosis in Hoechst 33342-treated HeLa cells and erlotinib-treated glioblastoma cells, respectively [47], [48]. Inhibition of autophagy by 3-methyladenine also accelerates rottlerin-induced caspase-independent apoptosis in HT1080 human fibrosarcoma cells [49].

**Figure 6:**
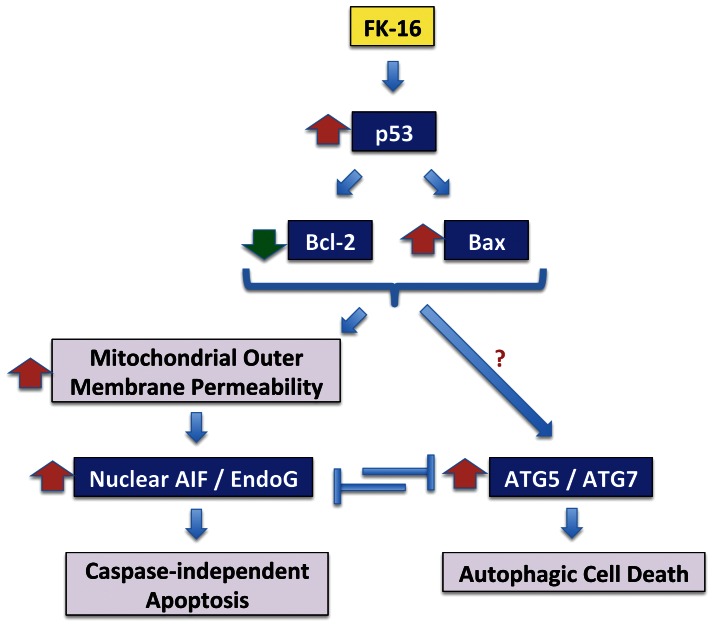
Schematic diagram showing the common regulation of caspase-independent apoptosis and autophagic cell death by the p53-Bcl-2/Bax cascade and their reciprocal regulation induced by FK-16 in colon cancer cells.

Identification of anticancer peptides from the existing AMP database is an efficient approach for the development of novel cancer therapeutics. In this study, FK-16, a fragment of LL-37, exhibits a better anticancer activity than the full-length peptide. FK-16 also engages an additional cell death pathway, namely, autophagic cell death. The shortened length of FK-16 may also reduce the production cost associated with peptide synthesis. Taken together, the anticancer peptide FK-16 induces AIF/EndoG-dependent apoptosis and autophagic cell death via the common p53-Bax/Bcl-2 cascade in colon cancer cells (Fig. 6). Our data also unveils a novel reciprocal regulation between these two caspase-independent cell death pathways.

## Materials and Methods

### Peptides

The synthetic LL-37 (LLGDFFRKSKEKIGKEFKRIVQRIKDFLRNLVPRTES), FK-16 (FKRIVQRIKDFLRNLV) and scrambled FK-16 (DQVLRFRNFRIKLVKI) peptides with purity >95% were purchased from Invitrogen (Carlsbad, CA, USA).

### Cell Culture and Cell Viability Assay

The human colon cancer cell lines HCT116 and LoVo were obtained from the American Type Culture Collection (Manassas, VA, USA). Bax^−/−^ and Bax^+/−^ HCT116 cells were generated by gene targeting [50] and provided by Prof. Bert Vogelstein (Ludwig Center at Johns Hopkins). The human hormal colon mucosal epithelial cell line NCM460 was acquired from INCELL Corporation. Cells were maintained in their respective recommended culture media, supplemented with 10% fetal bovine serum, 100 U/mL penicillin, and 100 µg/mL streptomycin at 37°C in a humidified atmosphere of 5% CO_2_ and 95% air. Cell viability was measured by MTT [3-(4,5-dimethylthiazol-2-yl)-2,5-diphenyltetrazolium bromide] assay. In brief, 5000 cells were plated per well in 96-well plates. After treatment, MTT solution dissolved in the culture medium at the final concentration of 0.5 mmol/L was added to each well and the plates were incubated for another 4 h. Dimethyl sulfoxide was then added to solubilize MTT tetrazolium crystal. Finally, the optical density was determined at 570 nm using a Benchmark Plus microplate reader (Bio-Rad, Hercules, USA).

### Quantitation of Phosphatidylserine Externalization

For measurement of phosphatidylserine externalization, treated cells were re-suspended in staining buffer containing propidium iodide and annexin V-fluorescein isothiocyanate (Invitrogen, USA). After incubation for 15 min in dark, double-labeled cells were analyzed by the FACSCalibur System and CellQuest program.

### Nuclear Protein Extraction and Western Blot

The isolation of nuclear and cytosolic proteins was performed using NucBuster Protein Extraction Kit (EMD Biosciences, Darmstadt, Germany) according to the manufacturer’s protocol. For isolation of whole-cell protein, cells were harvested in radioimmunoprecipitation buffer containing proteinase and phosphatase inhibitors. Equal amount of proteins were resolved by SDS–PAGE followed by a standard immunoblotting procedure.

### Immunofluorescence for AIF, EndoG and LC3

Cells grown on coverslips were fixed with 4% (v/v) paraformaldehyde for 30 min. The cells were then covered with 10% (v/v) goat serum for 60 min at room temperature followed by incubation with diluted primary antibody at 4°C overnight. Cells were then probed with Alexa Fluor 488 secondary antibodies (Invitrogen). Fluorescent signals were detected using a confocal fluorescence microscope (Nikon EZ-C1, Nikon, Tokyo, Japan).

### Bcl-2 Overexpression

The Flag-Bcl-2 expression vector (Addgene plasmid 18003) was obtained from Addgene, deposited by Dr. Clark W. Distelhorst (Case Western Reserve University, Cleveland, OH). Flag-Bcl-2 and the control vector pCMV-Tag2B were amplified using TOP10 competent *E. coli* (Invitrogen) and purified on QIAGEN Midi columns. Purified plasmids were transfected into HCT116 cells using Lipofectamine 2000 reagent (Invitrogen).

### RNA Interference

The expression of p53, AIF, EndoG, Atg5 and Atg7 were lowered using pre-designed target-specific small interference RNA (siRNA) molecules purchased from Qiagen (Valencia, CA, USA). Two hundred picomoles of gene-specific or control siRNA was transfected into cells at 40%–60% confluence using Lipofectamine™ 2000 reagent (Invitrogen).

### Detection of Acidic Vesicular Organelles (AVOs) by Acridine Orange Staining

Acridine orange (Sigma) was added at a final concentration of 1 µg/ml for a period of 15 min at 37°C. The stained cells were then washed twice with phosphate-buffered saline (PBS). Detection of fluorescence signal was performed with a fluorescence microscope (Nikon, TS-100-F) equipped with a 450–490-nm band-pass blue excitation filter, a 515-nm long-pass barrier filter, and a digital camera (Nikon DS-5Mc).

### Transmission Electron Microscopy

After treatment, cells were harvested by trypsinization, washed twice with PBS, followed by an overnight fixation with ice-cold glutaraldehyde [2.5% in 0.1 M cacodylate buffer (pH 7.4)] at 4°C. Samples were then washed by 0.1 M cacodylate buffer and then kept in 1% (wt/vol) osmium tetroxide at room temperature for 30 min. Afterward, cells were embedded in 2% (w/v) agar, which was then cut into 1 mm^3^ blocks. Agar blocks of fixed cells were immersed in a graded ethanol series from 30% to 90%, followed by three changes of absolute ethanol. Dehydrated agar blocks were then infiltrated by 33% and 66% Möllenhaucr’s resins in propylene (1.5 h each). Finally, the material was embedded in 100% resin and polymerised in an oven at 60°C for 24 h. Ninety nanometer-thick sections were cut, and then stained with saturated uranyl acetate for 30 min and lead citrate for 20 min. Demineralized sections were observed using a JEOL 100SX (Philips) transmission electron microscope at an accelerating voltage of 80 kV.
