# Correction: FK-16 Derived from the Anticancer Peptide LL-37 Induces Caspase-Independent Apoptosis and Autophagic Cell Death in Colon Cancer Cells

**DOI:** 10.1371/journal.pone.0131750

**Published:** 2015-06-25

**Authors:** Shun X. Ren, Jin Shen, Alfred S. L. Cheng, Lan Lu, Ruby L. Y. Chan, Zhi J. Li, Xiao J. Wang, Clover C. M. Wong, Lin Zhang, Simon S. M. Ng, Franky L. Chan, Francis K. L. Chan, Jun Yu, Joseph J. Y. Sung, William K. K. Wu, Chi H. Cho

The second author’s name is spelled incorrectly. The correct name is: Jing Shen.
